# Infectious prions in brains and muscles of domestic pigs experimentally challenged with the BSE, scrapie, and CWD agents

**DOI:** 10.1128/mbio.01800-25

**Published:** 2025-08-18

**Authors:** Francisca Bravo-Risi, Fraser Brydon, Angela Chong, Kane Spicker, Justin J. Greenlee, Glenn Telling, Claudio Soto, Sandra Pritzkow, Marcelo A. Barria, Rodrigo Morales

**Affiliations:** 1 Department of Neurology, The University of Texas Health Science Center at Houston 12340, Houston, Texas, USA; 2 Doctorado en Ciencias con Mención en Materiales Funcionales, Universidad Bernardo O'Higgins 28042, Santiago, Chile; 3 National CJD Surveillance Centre for Clinical Brain Science, University of Edinburgh 3124, Edinburgh, Scotland, United Kingdom; 4 Virus and Prion Research Unit, National Animal Disease Center, USDA, Agricultural Research Service 57837 , Ames, Iowa, USA; 5 Prion Research Center, Department of Microbiology, Immunology and Pathology, Colorado State University 3447, Fort Collins, Colorado, USA; 6 Centro Integrativo de Biología y Química Aplicada (CIBQA), Universidad Bernardo O'Higgins 28042, Santiago, Chile; Georgia Institute of Technology, Atlanta, Georgia, USA

**Keywords:** prion, pigs, chronic wasting disease, bovine spongiform encephalopathy, scrapie, PMCA, muscles, zoonotic infections

## Abstract

Experimental studies suggest that animal species not previously described as naturally infected by prions are susceptible to prion diseases affecting sheep, cattle, and deer. These interspecies transmissions may generate prions with unknown host ranges. Pigs are susceptible to prions from different origins, including deer chronic wasting disease (CWD), sheep scrapie, and bovine spongiform encephalopathy (BSE). Here, we studied prions in brains and muscles from pigs previously infected with these different prion sources. Specifically, we measured the total prion protein (PrP) and PK-resistant PrP by western blot. Seeding activity in these tissues was evaluated using the protein misfolding cyclic amplification (PMCA) technique. We found that BSE-infected pigs contained substantially more seeding competent prions compared with those infected with CWD and scrapie. Moreover, the zoonotic potential of porcine-BSE prions seems to be relevant, as both brains and muscles from BSE-infected pigs induced the misfolding of the human prion protein *in vitro*. This study helps to understand the potential fate of naturally existing prion strains in a relevant host and calls for caution considering the co-existence between feral swine and other prion-susceptible animal species.

**IMPORTANCE:**

Prions (PrP^Sc^) are proteinaceous, infectious pathogens responsible for prion diseases. Some livestock are highly susceptible to prion diseases. These include cattle (bovine spongiform encephalopathy, BSE), sheep and goat (scrapie), and cervids (chronic wasting disease, CWD). Unfortunately, BSE has been reported to be naturally transmitted to humans and other animal species. Domestic pigs, a relevant livestock animal, have not been reported to be naturally affected by prions; however, they are susceptible to the experimental exposure to BSE, scrapie, and CWD prions. Given the widespread consumption of porcine food products by humans, we aimed to evaluate the levels of pig-derived BSE, scrapie, and CWD prions from experimentally challenged domestic pigs in brain and meat cuts (leg, cheek meat, skirt meat, and tenderloin). We detected pig-adapted prions in the brains and some muscles of these animals. Additionally, we evaluated the *in vitro* compatibility between pig prions and the human prion protein (as a surrogate of zoonosis). Our results show that only pig-derived BSE prions were able to induce the misfolding of the cellular human prion protein. This data highlights the consequences of prion spillovers to other animal species and their potential availability to humans.

## INTRODUCTION

** **Prions are proteinaceous elements responsible for prion diseases (1). Animal prionopathies can acquire epizootic proportions in certain cases, as those associated with scrapie affecting sheep and goats, bovine spongiform encephalopathy (BSE), and chronic wasting disease (CWD) from cervids (2–5). Although strict measures were able to control the spread of BSE and scrapie, CWD still remains a problem considering that it also affects free-ranging animals (6–12). Another relevant problem linked with these animal prion diseases involves their host range. So far, only BSE has been shown to naturally infect other animal species, including ungulates, primates, felines, and humans (13–17). Experimentally, classical BSE is able to propagate in a wide variety of other animal species, demonstrating a wide host range (17–24). This also suggests that classical BSE may have caused interspecies subclinical prion infections that were never identified (25–28). On the contrary, scrapie seems to have a comparably narrower host range compared with BSE, and no human transmission of this animal prionopathy has ever been described (12, 29–31). Nevertheless, the case of scrapie is more complex considering the wide array of prion strains affecting sheep and goats, each one of them with specific interspecies penetrance as experimentally evaluated using mouse bioassays and *in vitro* prion replication techniques (22, 32–38). A scenario similar to scrapie is found for CWD: no transmissions to humans have been reported, and a wide array of strains has been recorded (39–45). Prion strains responsible for CWD also display differential host ranges, although these properties have been just partially identified (46–50). Although direct transmission of BSE, scrapie, and CWD to humans is an obvious concern, their propagation in other sympatric animal species could modify the host range potential of the original infectious agent, increasing their zoonotic potentials (51).

 In terms of animal species susceptible to prions, pigs are of particular interest. Pig-derived products constitute the second most common meat type consumed by humans (52, 53), and it is projected that consumption will increase by 8% by 2033 (52). In addition, by-products of the pig meat industry (blood, plasma, carcasses, hides, hoofs, heads, manure, offal, viscera, bone, fat, and meat trimmings) are also used for the production of cosmetics, biofuels and biomass-based diesel, fertilizers, medical and pharmaceutical applications, diagnostics and biotechnology (reagents), and human consumption (e.g., lard, sausage ingredients, black pudding, gelatin, jelly, hemoglobin, and other edible by-products such as cracklings, ears, heart, kidney, liver, omentum, skirt, spleen, stomach, tail, and tongue). Pig derivatives are also used as protein sources for pet and livestock (cows, sheep, and goats) feed (54, 55), events that are relevant for potential interspecies prion transmissions. Considering all uses of pig products and their diverse interactions with humans, it is relevant to understand whether this animal species is susceptible to prions from naturally infected sympatric species. It is also important to understand whether prions emerging from these events present any potential threat for human health.

While natural cases of prion diseases in domestic pigs have not been reported, some groups have experimentally explored their susceptibility to classical (C-type) BSE, scrapie (classical and atypical), CWD, and sheep-derived BSE. These experiments have been done in the relevant host (domestic pigs), transgenic mice over-expressing the porcine prion protein, and *in vitro* assays (20, 26, 36, 56–61). Domestic pigs simultaneously challenged with BSE by three routes (intracranially, intravenously, and orally) demonstrate efficient transmission of the disease that was linked with the presence of infectious prions in multiple tissues (20, 62). As expected, the transmission of BSE prions to pigs was route-dependent as animals challenged by the oral route resulted in subclinical infections (62). Interestingly, the biochemical properties of the porcine-adapted BSE prions were different compared with those of the original source, demonstrating the emergence of a new prion strain (63). A similar phenomenon was also observed after scrapie exposure to domestic pigs, although subclinical transmission in this case was observed for pigs injected by the intracerebral and oral routes (56). Experimentally challenged domestic pigs are also subclinically susceptible to CWD by oral and intracerebral routes, although with incomplete attack rates and long incubation periods (61). In this particular case, the biochemical features of the newly generated prions also differed from the original source (61). The study of the susceptibility of pigs to CWD is relevant considering a recent article describing the interaction between this particular infectious agent and feral swine in natural settings (64). In summary, previous evidence suggests that although domestic pigs are susceptible to BSE, scrapie, and CWD prions, these interactions are restricted by strong species barriers. Importantly, successful prion transmissions of prion disease into pigs result in new prion strains, a fact that is problematic considering the use of pig products in the manufacture of human and animal feed and other products relevant for human activities.

Here, we describe the *in vitro* propagation properties of porcine-adapted prions using a porcine-adapted version of the protein misfolding cyclic amplification (PMCA) technique. These analyses were performed in brain and muscle tissues from prion-infected domestic pigs. Muscles were evaluated in this study, considering their relevant use in human nutrition. Moreover, the same tissues were analyzed for their molecular compatibility with human forms of the prion protein using the PMCA assay. These results are useful to understand the potential fate of natural animal prions if spillover occurs into other animal species.

## RESULTS

### Characterization of total prion protein (PrP) and disease-associated prion protein (PrP^Sc^) in brains and skeletal muscles of prion-infected pigs

**Fig 1:**
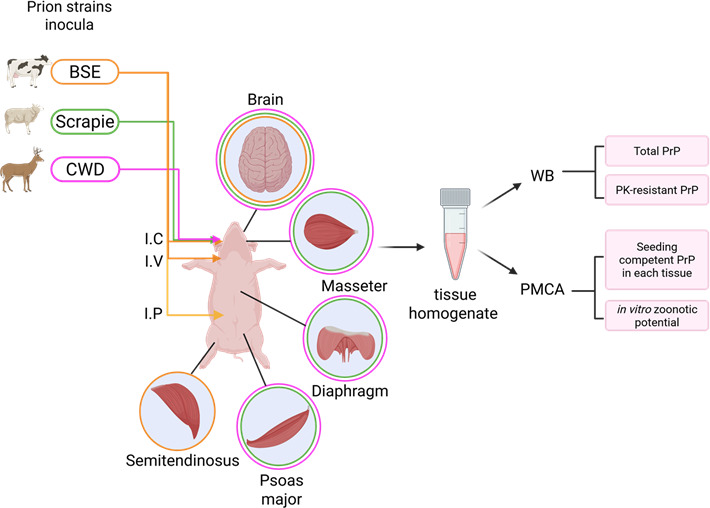
Experimental strategy. Brain (*n* = 3/inoculum) and skeletal muscle (*n_total_* = 21) specimens from domestic pigs experimentally inoculated with C-type BSE (yellow), scrapie (green), and CWD (pink) prions were interrogated for their prion content using western blotting and PMCA. The routes of inoculations in pigs were intracerebral (I.C), intravenous (I.V), and intraperitoneal (I.P) for the BSE prions, while scrapie and CWD prions were administered by the I.C. route only. The specific skeletal muscles assessed were semitendinosus (pig-adapted BSE), masseter, diaphragm, and psoas major (pig-adapted scrapie and CWD). The PMCA protocol used in this study was optimized to replicate pig PrP^Sc^, as described (64). Finally, the zoonotic potential of the pig-adapted prions was evaluated by PMCA reactions using the 129M and 129V human PrP^C^ variants as substrate. This figure was generated using Biorender.com.

**Fig 2:**
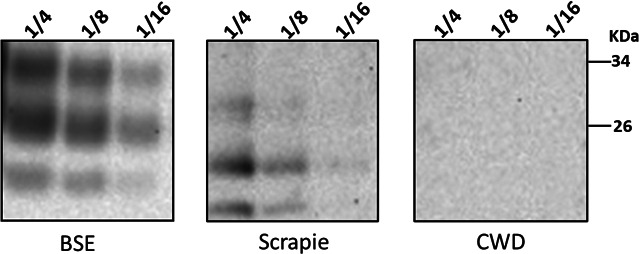
Levels of PK-resistant PrP^Sc^ in the brains of domestic pigs experimentally challenged with classical BSE, scrapie, and CWD prions. Representative Western blot of PK-resistant PrP levels from samples 310 (pig-BSE), 371 (pig-scrapie), and 423 (pig-CWD). Brain homogenates (BH) were treated with PK (50 µg/mL), then diluted in PBS, and evaluated by Western blot. The specimens from pigs exposed to BSE were performed in duplicates, and those inoculated with scrapie and CWD were performed in triplicates. Numbers at the top of the panels represent the dilution of BH used. The name at the bottom of each panel depicts the prion strain used as inoculum for the domestic pigs. The number at the right represents molecular weight markers (in KDa).

A set of nine brains and 21 skeletal muscle specimens from domestic pigs experimentally challenged with C-type BSE (20), sheep (ARQ homozygous) scrapie (56), and white-tailed deer (PrP 96 G/G) CWD (61) prions was analyzed (Fig. 1 and Materials and Methods). First, we interrogated these samples for their total porcine prion protein (PrP) content using western blotting (WB). PrP was detected in all brain samples, with higher levels in the brains from swine exposed to C-type BSE. In contrast, PrP detection was not detected in all the different muscle-type tissues using this technique (Fig. S1). Furthermore, we submitted the same specimens to a proteinase K (PK) treatment to assess the presence of PK-resistant PrP. Our results consistently demonstrate the presence of PK-resistant prions in brain tissues from domestic pigs exposed to C-type BSE in all replicates (Fig. 2). Protease-resistant prion proteins were detected in just 1/3 brain sample (sample 371) from the scrapie-infected pigs (Fig. 2). On the contrary, no detection of PK-resistant PrP was retrieved from any of the pigs challenged with the CWD agent (Fig. 2; Fig. S2). Serial dilutions of representative samples from each group demonstrated that the load of PK-resistant PrP was higher in pigs infected with C-type BSE prions (Fig. 2). In summary, our observations on the levels of PK-resistant PrP in prion-infected pigs suggest higher prion titers in swine inoculated with BSE, followed by scrapie-infected animals.

### *In vitro* seeding efficiency of PrP^Sc^ in the brains from pigs infected with BSE, scrapie, and CWD prions

**Fig 3:**
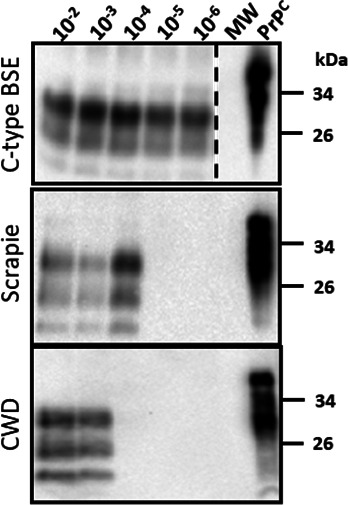
Identification of seeding-competent pig-derived BSE, scrapie, and CWD prions in brain tissues by PMCA. Representative western blots depicting the prion seeding activity (third PMCA round) in brain tissues (samples 310 [pig-BSE], 371 [pig-scrapie], and 399 [pig-CWD]). The number at the top of the panels represents the final concentration of the brain homogenates submitted to the PMCA substrate (ranging from 10^−2^ to 10^−6^ g/mL). Each sample was analyzed in triplicate. The pig-adapted scrapie and CWD prions were detected by incubating the membranes with the mAb 8H4 at a dilution of 1:5,000. The numbers at the right represent molecular weight markers (in KDa). The MW represents the EZ-Run molecular weight ladder (Fisher Scientific, Waltham, MA, USA). “PrP^C^” denotes brain extracts from Tg002 mice (expressing the pig version of PrP^C^) not treated with PK and used as an electrophoretic mobility and antibody reactivity control. The dashed lines represent sites where the membrane was cropped/edited. All images come from the same membrane.

**Fig 4:**
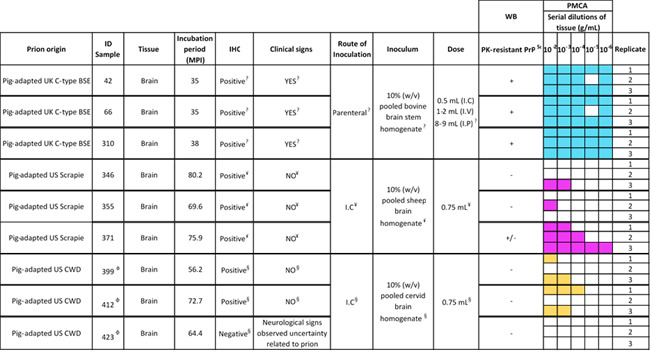
Individual results of the seeding activities from pig-derived BSE, scrapie, and CWD prions from domestic pigs’ brains. This figure shows the raw data of PMCA analyses in each brain specimen. Three replicates were tested in each case, at different dilutions. Other information, relevant for this Figure, is presented as follows: ^ǂ^ Ryder, S. J., Hawkins, S. A. C., Dawson, M., & Wells, G. A. H. (20). The neuropathology of experimental bovine spongiform encephalopathy in the pig. *Journal of Comparative Pathology*, *122*(2–3), 131–143. https://doi.org/10.1053/jcpa.1999.0349. ^¥^ Greenlee, J. J., Kunkle, R. A., Smith, J. D., & Greenlee, M. H. W. (56). Scrapie in Swine: a Diagnostic Challenge. *Food Safety*, *4*(4), 110–114. https://doi.org/10.14252/foodsafetyfscj.2016019. ^§^ Moore, S. J., West Greenlee, M. H., Kondru, N., Manne, S., Smith, J. D., Kunkle, R. A., Kanthasamy, A., & Greenlee, J. J. (61). Experimental transmission of the chronic wasting disease agent to swine after oral or intracranial inoculation. *Journal of Virology*, JVI.00926-17. https://doi.org/10.1128/JVI.00926-17. ^£^ Observed in some replicates. ^Φ^ Samples 399, 412, and 423 in this study are from animals 26, 28, and 27 from the reference (61) study, respectively.

As previously described, we adapted a PMCA system to specifically detect porcine-derived prions with high sensitivity (64). Here, we used this system to evaluate the seeding capacity of brain samples from pigs infected with BSE, scrapie, and CWD. This analysis might provide relevant information on the replication potential of these porcine-adapted prions. The PMCA reactions for each tissue were performed in serial dilutions ranging from 10^−2^ to 10^−6^ g/mL with the goal to assess the prion amplification limit in each case. Each sample was tested in triplicate, and the partially resistant core protein fragments (PrP^Sc^) were visualized by WB after three rounds of PMCA. Our results demonstrate an efficient prion replication for the pig-adapted BSE prions, as all the samples and dilutions tested provided positive signals (Fig. 3 and 4). Prion amplification was also identified in the brains of all pigs treated with scrapie prions; however, the amplification efficiency in this group of specimens was lower compared with that observed for their BSE-derived counterparts. Interestingly, the porcine-adapted scrapie sample 371 showed significantly higher levels of amplification compared with the other two specimens from the same group (10^−6^ vs. 10^−2^ and 10^−3^ g/mL). Importantly, these data were consistent with the fact that this was the only scrapie-derived sample displaying PrP^Sc^ directly via WB (Fig. 2 to 4) and with the fact that this sample provided a 4.0 OD value on IDEXX EIA compared to 3.3 for sample #346 and 2.1 for #355 (56). In contrast, brain samples from only 2/3 pigs infected with CWD showed prion replication, with a lower maximum efficiency compared with the other two groups (10^−3^ and 10^−4^ g/mL, Fig. 3 and 4). It is important to mention that all samples used in this study were previously reported to contain prion deposits in their brains by IHC, with the sole exception of the CWD-negative sample in PMCA, which was selected considering the presence of clinical signs (Fig. 4). Overall, this data confirms the presence of porcine-adapted prions in all groups tested in this study, although a higher amplification efficiency was observed for the porcine-adapted BSE prions when compared with their scrapie and CWD counterparts.

### Prion seeding activity in skeletal muscles of experimentally infected domestic pigs

**Fig 5:**
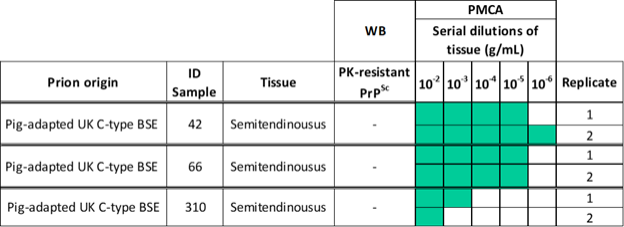
Individual results of the *in vitro* seeding activity of skeletal muscle specimens from domestic pigs experimentally challenged with classical BSE prions. This figure shows the raw data of PMCA analyses in each muscle specimen for pigs infected with BSE prions. Two replicates were tested in each case, at different dilutions.

**Fig 6:**
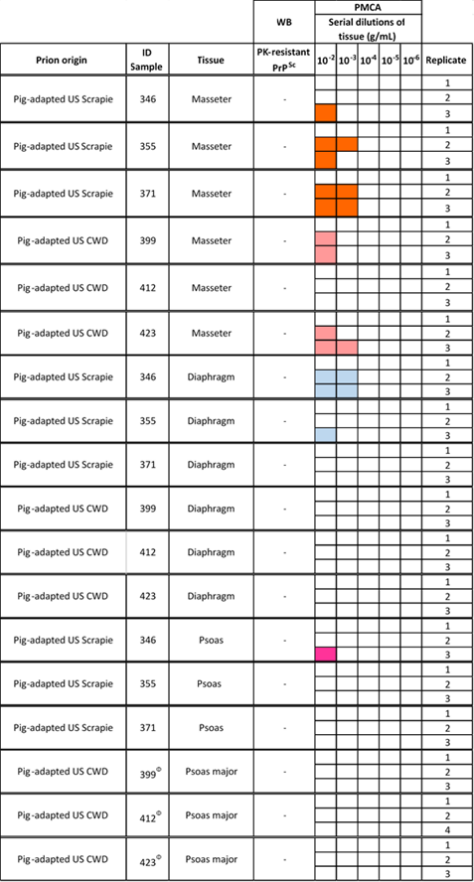
Individual results of the *in vitro* seeding activity of skeletal muscle specimens from domestic pigs experimentally challenged with scrapie and CWD prions. This figure shows the raw data of PMCA analyses in each muscle specimen for pigs infected with either scrapie or CWD prions. Three replicates were tested in each case, at different dilutions. Other information, relevant for this figure, is presented as follows: ^Φ^ Samples 399, 412, and 423 in this study are from animals 26, 28, and 27 from the reference (61) study, respectively.

Pig muscles are relevant considering their use in human nutrition. For that reason, we evaluated the presence of seeding-competent prions in skeletal muscles from pigs infected with BSE, scrapie, and CWD. Importantly, muscles from the same brain donors described above were tested. Due to sample availability, different muscle types were tested in each group (Fig. 1). Specifically, we screened the semitendinosus muscles from pigs inoculated with BSE, and masseter, diaphragm, and psoas major muscles from animals inoculated with scrapie and CWD prions (Fig. 1, 5, and 6). Our results demonstrated relatively high levels of pig-BSE prions in the semitendinosus muscles of two pigs (IDs 42 and 66), as seeding activity was detected at tissue concentrations ranging from 10^−5^ to 10^−6^ g/mL. Interestingly, the detection of the infectious agent in the muscle from the remaining animal (ID 310) reached maximum detection at considerably higher concentrations compared to the other two specimens (10^−2^ to 10^−3^ g/mL) (Fig. 5). These data suggest that PrP^Sc^ is relatively abundant in the muscles of BSE-infected pigs, although their levels may vary between individuals.

**Fig 7:**
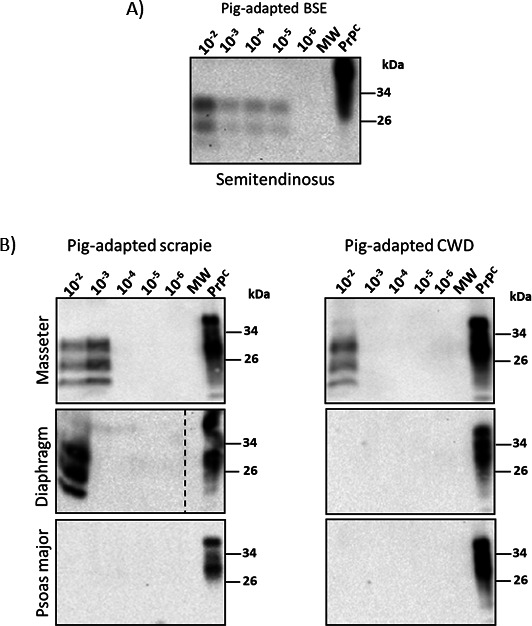
*In vitro* conversion efficiency of pig PrP^C^ templated by pig-derived BSE, scrapie, and CWD prions in skeletal muscles. Representative Western blots depict PrP^Sc^ detected in muscle specimens. The tissues were serially diluted (10^−1^ to 10^−5^ g/mL) and submitted to three PMCA rounds. (**A**) Semitendinosus muscle homogenates from a pig inoculated with C-type BSE interrogated in duplicate. (**B**) Masseter, diaphragm, and psoas major homogenate tissues from domestic pigs experimentally exposed to scrapie and CWD prions were analyzed in triplicate. The numbers at the top of the panels represent the final dilutions of the samples in the pig PMCA substrate. The numbers at the right represent molecular weight markers (in KDa). The MW represents the EZ-Run molecular weight ladder (Fisher Scientific, Waltham, MA, US). “PrP^C^” denotes brain extracts from Tg002 mice (expressing the pig version of PrP^C^) not treated with PK and used as an electrophoretic mobility and antibody reactivity control. The dashed lines represent sites where the membrane was cropped/edited.

Next, we evaluated the prion seeding activity of muscles from pigs infected with scrapie and CWD (Fig. 6 and 7). The results showed detection of pig-scrapie prions in the masseter muscles of all three animals. In contrast, the seeding activity of pig-CWD prions in this muscle type was achieved in only two samples (IDs 399 and 423). The detection of prions in diaphragm and psoas major muscles was much lower compared to the masseter muscle. Specifically, detection was achieved in samples from just two (diaphragm) and one (psoas major) pigs infected with scrapie. No detection in any of these two muscles was achieved for pigs infected with CWD. When positive, these muscle samples supported prion amplification only at high concentrations (10^−2^ to 10^−3^ g/mL), suggesting lower concentrations of prions in the samples (Fig. 6 and 7).

 In summary, these results demonstrate that pigs infected with BSE, scrapie, and CWD harbor prions in their muscles, although their quantities and distribution vary depending on the prion source. Nevertheless, considering the differential routes of exposure between the groups (BSE vs. scrapie and CWD), we cannot discard this as a potential source of the differential results observed in these analyses.

### Evaluation of the zoonotic potential of brains and muscles derived from prion-infected pigs

**Fig 8:**
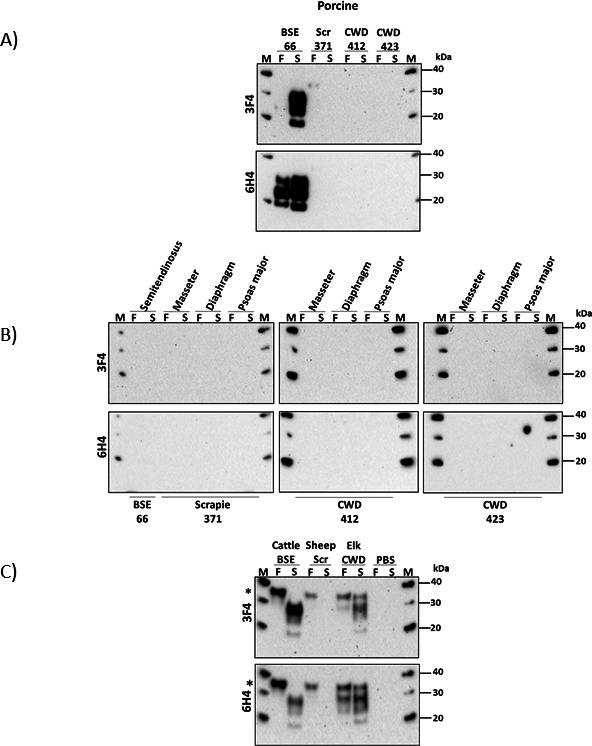
*In vitro* misfolding of human prion protein (129M) seeded with abnormal prion proteins derived from domestic pigs experimentally inoculated with the classical BSE, scrapie, and CWD agents (Site 1). (**A**) Humanized PMCA reactions using brain homogenates from swine inoculated with C-type BSE, scrapie (scr), and CWD as inocula. (**B**) Humanized PMCA reactions using muscle homogenates from swine inoculated with C-type BSE, scrapie (scr), and CWD as inocula. (**C**) PMCA controls for these experiments included reactions seeded with C-type BSE, classical scrapie, and CWD (Elk) of known seeding activity. PBS was also used as an additional technical control (37). F (frozen) represents aliquots of each reaction collected before amplification and stored at −80 °C for comparison. S (sonicated) denotes samples harvested post-amplification. PMCA products were detected using the mAb 3F4, which recognizes the amino acid sequence between 109 and 112 of the human prion protein and does not cross-react with porcine PrP. The contribution of the inocula to the 3F4-visualized signals was assessed by re-incubating these membranes with the mAb 6H4 (detecting both human and porcine prion sequences). M denotes the molecular weight marker (in KDa). (*) Asterisks indicate undigested PrP material.

Next, we evaluated the zoonotic potential of pig brains and muscles using the PMCA technique. This was achieved using a subset of tissues (IDs 42, 66, 310, 371, 412, and 423) and a PMCA protocol specifically designed for this purpose (37). These experiments were conducted using one PMCA round, as this was expected to best mimic initial interspecies conversions, as previously described (37). In addition, these experiments were conducted in two different laboratories (Site 1 and Site 2) to assess the reproducibility of the results. In the first site (Site 1), we used the 129 MM polymorphic version of human PrP as PMCA substrate, considering its higher propensity for zoonotic transmissions (65, 66). PMCA products were visualized using a human-specific anti-prion antibody (3F4) to discard the presence of the original inocula in the readouts. The results show that conversion of the human prion protein was induced only by the brain samples of the BSE-inoculated pigs. The brain samples from scrapie- and CWD-infected pigs, and muscle samples from all three experimental groups, did not induce the misfolding of the human prion protein in this specific PMCA system (Fig. 8A and B). Importantly, this assay was controlled by positive controls including PMCA reactions seeded with either BSE and elk-CWD prions previously shown to misfold the human prion protein (37). Negative controls included reactions primed with PBS (Fig. 8C). Probing the same membranes with the 6H4 antibody, which detects prions from different animal species, provided complementary information of the presence of the original prion seed included in each reaction. In summary, our results suggest that only BSE-derived pig prions exhibit molecular compatibility with the human prion protein in terms of *in vitro* propagation.

**Fig 9:**
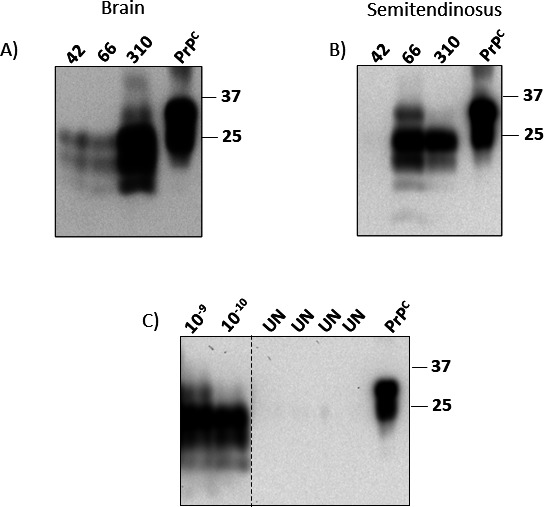
*In vitro* misfolding of human prion protein (129M) seeded with abnormal prion proteins derived from domestic pigs experimentally inoculated with the BSE, scrapie, and CWD agents (Site 2). (**A**) Humanized PMCA (129M) reactions seeded with brain homogenates from three pigs (IDs 42, 66, and 310) inoculated with C-type BSE. (**B**) Humanized PMCA (129M) reactions seeded with muscle homogenate from three pigs (IDs 42, 66, and 310) inoculated with C-type BSE. (**C**) Technical controls consisted of seeded reactions with serial dilutions (10^−9^ and 10^−10^) from the brain homogenate of a vCJD individual. Additionally, unseeded (UN) PMCA reactions were included. PMCA products were detected using the mAb 3F4 (1:5,000) after PK digestion. Numbers at the right of each panel represent molecular weight markers (in KDa). “PrP^C^” denotes brain extracts from Tg-HuMM mice (expressing the 129M version of human PrP) not treated with PK and used as an electrophoretic mobility and antibody reactivity controls. The dashed line represents cropped/edited membranes.

 The results obtained above were largely confirmed on a second site (Site 2) that consistently showed the misfolding of human prions by the BSE-porcine adapted brain materials (Fig. 9A). Moreover, additional PMCA reactions conducted in this site demonstrate that muscle samples from 2/3 of these pigs were able to induce the misfolding of the human 129M PrP (Fig. 9B). Additional analyses of the same tissues, and those from scrapie- and CWD-infected pigs, showed no effect in the misfolding of the 129V polymorphic version of the human prion protein (Fig. S3).

## DISCUSSION

In this study, we evaluated the presence and relative quantities of prions in brains and muscles from pigs infected with three different prion isolates: C-type BSE, scrapie, and CWD. Our findings confirm previous reports by demonstrating the presence of disease-associated prions in the brains of these animals (20, 56, 61). In addition, we evaluated the replication capacity of the prions present in these samples using the PMCA technique. The presence of prions in the brains of these prion-infected pigs appears to be variable depending on the original prion source. Although prion seeding activity was identified in the brains of pigs from all three groups, we found that those from animals infected with BSE contained the most active seeds. It is relevant to mention that most of the pigs infected with either scrapie or CWD did not display neurological signs linked with prion disease, yet prion seeding activity was still detected in their brains. The Moore et al. publication (61) states the presence of sparse PK-resistant PrP accumulation in the brain of CWD-infected pigs, suggesting a low amount of PrP^Sc^ in these samples. To improve the detection, the authors treated brain extracts with a sarkosyl/ultracentrifugation protocol and loaded at 100 mg of brain tissue for detection via western blots. Due to the limited amount of sample available for the present study, we did not enrich the sample and loaded 2 mg of brain tissue on western blots. This might have affected the detection of the PrP signals using this method (Fig. 2; Fig. S2). The low amount of PrP^Sc^ in these samples was further confirmed by PMCA, as seeding activity was present in just some replicates (Fig. 4), mostly at low dilutions. Nevertheless, the identification of subclinical prion infection in these brains is consistent with previous studies suggesting the same when bioassays in transgenic mice expressing the porcine PrP^C^ were used in the BSE and CWD transmissions (26, 56). The fact that subclinical prion infection was also identified in pigs orally exposed to BSE and CWD (56, 61, 62) suggests that transmissions in natural scenarios are possible and an eventual adaptation of the agent in pigs (e.g., considering the cannibalistic behavior of wild pigs) is plausible.

In addition to the prion content in brains, we evaluated the anatomical distribution of PrP^Sc^ in several skeletal muscles with the goal of understanding the tropism of the adapted prions in these tissues. The specific muscles tested included semitendinosus (leg), masseter (cheek meat), diaphragm (skirt meat), and psoas major (tenderloin). These pork cuts are commonly consumed by people and are commercially available. Unfortunately, the muscle tested for the BSE-infected group (semitendinosus) differed from those analyzed in the scrapie and CWD groups (masseter, diaphragm, and psoas) due to the availability of archived materials. Regardless, these samples are useful to understand whether prions in pigs accumulate in muscles that might be available for human consumption. Our results indicate that the muscles collected from the BSE-infected pigs contain higher levels of seeding-competent prions compared with those in other groups. Nevertheless, the same cautions regarding the routes of administration of BSE materials in pigs explained above must be considered when interpreting this data. The comparison between scrapie and CWD inoculated pig groups, that were inoculated with the same quantities of infectious materials and the same route, and where the same tissues were collected, allows us to make more accurate associations. In summary, our findings demonstrate that muscles closer to the animal head (masseter) supported a better seeding activity compared to those located at the thorax (diaphragm) and hind levels (psoas major). One explanation for this could be explained by the centrifugal spreading of prions, which agrees with the findings by Headman and colleagues (58) describing the detection of PrP^Sc^ in upper muscles of clinically affected pigs inoculated intracerebrally with sheep-BSE prions. In that study, two swine harbored PrP^Sc^ in muscles associated with the eyes (oculomotor muscle), while prions were identified in the semitendinosus muscle of just one animal. Recently, the same group analyzed the peripheral tissues of these pigs by PMCA, detecting seeding activity in the oculomotor muscle in all the animals exposed to the BSE-derived agent in dilutions as low as 10^−4^ (67). Due to the limited amount of muscle samples from the pigs inoculated with BSE, we were unable to perform a detailed anatomical analysis. However, we showed that the semitendinosus muscle, which is located at the hind limbs, contained relevant levels of seeding competent PrP^Sc^. The relevantly abundant presence of prions in this particular group of pigs could be due to the fact that they were peripherally exposed to the agent, or by the peripheral tropism of BSE prions as it has been observed for the transmission of this particular prion strain to multiple other animal species (62, 68–80). The data describing higher quantities of seeding-competent prions in the brains of BSE-infected pigs needs to be interpreted with caution. As described, BSE-infected pigs received infectious materials at higher doses and through three simultaneous routes of administration compared to the animals exposed to the scrapie and CWD agents (20, 56, 61). If we restrict these comparisons to the latter two groups (scrapie- and CWD-infected pigs) that were exposed to the same prion doses (750 µL of 10% [w/v] pooled sheep and cervid brain homogenate, respectively), and through the same route of inoculation (intracerebral), we found that pig-adapted scrapie prions exhibited a higher amount of seeding-competent PrP^Sc^ in both brain and muscle tissues compared with their CWD-exposed counterparts. The differences in seeding capacities and tropisms between these newly generated porcine prions can be explained in the generation of different prion strains in each case. Nevertheless, it is also relevant to consider that these studies used unique scrapie and CWD strains from the many described in experimental and natural settings (31, 33, 40, 41). Along this line, future studies should focus on the differential susceptibilities experienced by pigs when exposed to different variants associated with these animal prionopathies.

Importantly, our studies evaluating zoonotic potentials showed that pig-adapted BSE prions were able to induce the misfolding of the human prion protein *in vitro*. These results were confirmed in two different laboratories, a fact that provides rigor to this study. Importantly, these analyses also show that muscles from these BSE-infected pigs may present a concern if introduced in the human food chain. Importantly, the zoonotic risk of these materials seems to be restricted to the 129M polymorphic version of the human prion protein, in line with the higher susceptibility of BSE to the population carrying this specific protein. Previous reports have shown that mice expressing the 129M human PrP^C^ variant (HuPrP-tg 650 and 340) exhibited a species barrier to BSE prions that was overcome in a first transmission passage (81). Similar outcomes were observed when pig-adapted BSE prions were inoculated in HuPrP-tg340 mice, although with incomplete attack rates (50%) in a first passage. Serial transmissions of this infectious material in the same mice resulted in complete attack rates and reduction in the incubation periods, suggesting that the infectious material successfully adapted in the new host (60). On the contrary, extensive evidence demonstrates that the species barrier between scrapie and CWD prions and humans is strong, if not absolute (39, 42, 82). However, the existence of different prion strains associated with these animal prionopathies urges for a systematic analysis including the whole pathogenic spectrum associated with them. In our study, scrapie- and CWD-adapted prions were not able to induce the misfolding of the human prion protein. However, considering the data from the porcine-BSE prions, we cannot conclude whether the lower load of prions in the CWD- and scrapie-porcine tissues was responsible for these results.

Some of the limitations of the current study have been already discussed. One of the issues involves the unknown prion infectivity titers of the BSE-, scrapie-, and CWD-bearing tissues used to infect pigs. This importantly limits comparisons between the groups. Prion infectivity titers can affect tissue tropisms, especially in subclinical transmissions (as prions may need additional time to reach target tissues). This could be considered an additional source of variability for the dissimilar presence of prions in muscles for the different animal groups included in this study. Another limitation involves the uneven testing of muscle tissues across the three groups and the lack of analysis of other muscles representing different anatomical distributions within the body. The latter may be relevant for understanding the tropism of prions induced by each injectate. Finally, the scrapie and CWD groups were treated with specific prion strains from many that have been described. This is relevant considering that different strains may adapt with different efficiencies into new hosts and result in infectious particles with unique host ranges. Future studies should focus on the above-mentioned limitations. However, we believe that the current study provides a solid background to justify these analyses.

In summary, our data shows the dynamic of animal prions when exposed to infectious pigs, as well as their distributions and zoonotic potentials. The data presented here may be relevant to understanding the fate of naturally existing prions in a sympatric animal species relevant for human consumption. This acquires importance considering a recent report describing the interaction between CWD and wild pigs in natural settings.

## MATERIALS AND METHODS

### Brain and muscle sample collection and preparation

A set of 30 prion-infected pig tissues was provided by Dr. Greenlee (56, 61) and the UK Animal and Plant Health Agency (20) and stored at –80 °C until used. The tested samples included brain (*n* = 9, three per inoculum) and muscles (*n* = 21) from nine swine experimentally exposed to BSE, scrapie, and CWD prions. Specifically, the skeletal muscle samples analyzed included semitendinosus muscles from BSE-inoculated pigs (parentally) (20), and masseter, diaphragm, and psoas major muscles were collected from scrapie- and CWD-intracranially inoculated domestic pigs (56, 61) (Fig. 1). The scrapie source for treating pigs was the brain tissue of an infected ARQ/ARQ sheep. The CWD prions used to treat pigs were obtained from a brain homogenate pool made from three white-tailed deer previously inoculated with a mix of brain homogenates from CWD-affected white-tailed deer, elk, and mule deer. The tissues were thawed on ice and homogenized at 10% (w/v) using cold PBS containing a freshly added cOmplete cocktail of protease inhibitors (Roche, Basel, Switzerland). Mechanical tissue disruption was performed using soft tissue homogenizing CK14 2 mL tubes containing 1.4 mm ceramic beads (Bertin Corp, Rockville, MD, USA) in a Precellys 24 dual homogenizer (Bertin instruments, MP biomedicals, Irvine, CA, USA) and stored at −80 °C until used. The tissues were diluted serially (10^−1^ to 10^−5^ g/mL) using cold PBS supplemented with cOmplete EDTA-free cocktail of protease inhibitors (Roche, Basel, Switzerland) and used as seed in the protein misfolding cyclic amplification (PMCA) reactions.

### Porcine PMCA

The PMCA was adapted to replicate the misfolding of the porcine prion protein. The PMCA substrate was prepared at 10% (w/v) using a pool of brains from male and female PoPrP-Tg002^(+/-)^ mice expressing the porcine version of PrP^C^ (56) and stored at −80 °C until used. The PMCA substrate was freshly supplemented with digitonin at a final concentration of 0.05% (v/v) (Invitrogen, Carlsbad, CA, USA) and 5 mM EDTA (Promega Corporation, Madison, WI, USA). Afterward, 10 µL of serial dilutions of brain and muscle tissues were independently added into 0.2 mL PCR tubes (Eppendorf, Enfield, CT, USA) containing 90 µL of the final PMCA substrate, and three 3/32″PTFE beads (Engineering Laboratories, Inc., Oakland, NJ, USA). As a PMCA control, serial dilutions (10^−3^ to 10^−12^ g/mL) of a PMCA-adapted material of known seeding activity were used as a seed and included in each reaction set. The PMCA-adapted material was originated from a pig-adapted scrapie PrP^Sc^ that was serially passaged in Tg002 substrate, as described (64). Four unseeded reactions were included as negative control in each PMCA reaction set. The PMCA products were evaluated in a third round of PMCA and visualized by western blotting. The analyses were performed in duplicate or triplicate as shown in Fig. 5 and 6.

### Proteinase K (PK) treatment

The samples used to analyze the PK-resistant PrP, the levels of PrP^Sc^, and the PMCA products (porcine PMCA) were treated with PK (Sigma-Aldrich, Saint Louis, MO, USA) at a final concentration of 50 µg/mL at 37 °C for 90 min and shaking at 450 rpm in an Eppendorf thermomixer. After digestion, the brain samples used to test the levels of PrP^Sc^ were submitted to serial dilutions (dilution factor 2, 4, 8, and 16) using PBS. Then, NuPAGE LDS sample buffer was added to all the reactions at a final concentration of 1X (Invitrogen, Carlsbad, CA, USA). The proteolytic reactions were finally stopped by heating them at 95 °C for 10 min in a heat block.

### Western blot

To visualize the total PrP present in pigs’ brains and muscles, each specimen’s homogenate was mixed with NuPAGE LDS sample buffer at a final concentration of 1X (Invitrogen, Carlsbad, CA, USA) and incubated at 95 °C for 10 min in a heat block. Samples treated and not treated with PK were fractionated using NuPAGE 12% Bis-Tris gels (Invitrogen, Carlsbad, CA, USA) with 1X NuPAGE MOPS SDS running buffer (Invitrogen, Carlsbad, CA, USA). The electrophoresis settings used were as described (83). The proteins were transferred into a nitrocellulose membrane (GE Healthcare Amersham, Chicago, IL, USA) using the Trans-blot Turbo Transfer System (Bio-Rad, Hercules, CA, USA) for 7 min at 2.5 A. The gel, membrane, and filters were soaked in a 1X Trans-blot Turbo Transfer Buffer (Bio-Rad, Hercules, CA, USA), and the blotting sandwich was assembled following the manufacturer’s recommendations. The membranes were blocked with 10% (w/v) non-fat milk prepared in washing buffer (PBS, 0.05% [v/v] Tween-20) for 15 min at room temperature (RT). The membranes containing samples with the pig-adapted BSE prions were immunolabeled with the primary monoclonal antibody (mAb) anti-prion protein 6H4, produced in mouse (Prionics, Zurich, Switzerland), which recognizes the amino acid sequence 144–152 (DYEDRYYRE) of the human prion protein. The membranes containing the pig-adapted scrapie and CWD prions were detected using the mAb produced in mouse clone 8H4 (Cat. # P0110-200 UL, Sigma-Aldrich, Inc., St. Louis, MO, USA), as the 6H4 antibody was commercially discontinued. The mAb 8H4 recognizes the human prion protein epitope between amino acids 145 and 180. The primary antibodies were incubated overnight (ON) at 4°C. The mAb 6H4 was prepared at a dilution 1:10,000, while the mAb 8H4 was prepared at a dilution of 1:10,000 and 1:5,000 depending on the lot used. Afterward, the membranes were incubated with polyclonal anti-mouse IgG (whole molecule)–peroxidase antibody produced in sheep (Cat. # A5906-1ML, Sigma-Aldrich, Saint Louis, MO, USA) at a 1:3,000 dilution for 60 min at room temperature. After each antibody incubation step, the membranes were rinsed three times for 10 min with washing buffer. The same buffer was used to dilute the antibodies. The membranes were developed using ECL (GE Healthcare Amersham, Chicago, IL, USA) following the manufacturer’s recommendations. The images were acquired in a BioRad dark chamber and processed using the Image Lab (Bio-Rad, Life Science, Hercules, CA, USA) and the Fiji (Image J) software (84).

### Evaluation of the zoonotic potential of porcine tissues using PMCA (Site 1)

This procedure was performed following the protocols described in our previous publication (37). Transgenic mouse brains expressing the human version of the prion protein (129M [85]) were homogenized using a glass-on-glass manual grinder in a conversion buffer made of PBS, 150 mM NaCl, 1% Triton X-100, and a complete protease inhibitor cocktail (cOmplete; Roche, Mannheim, Germany) to yield a final 10% w/v mixture. The homogenized tissue was cleared by using centrifugation (40 s at 805 × *g*). Then, the supernatant (i.e., human PMCA substrate) was aliquoted in 1.5 mL tubes and stored at −80 °C until used. The *in vitro* prion amplification reactions were conducted in a programmable Q-700 sonicator attached to a microplate titanium horn. Low molecular weight heparin at 100 µg/mL and EDTA was included in all the 1.5 mL tubes to a final concentration of 6 mM each. Elk meat homogenates were mixed with aliquots of human PMCA substrate in a final volume of 120 µL in PCR tubes at a 1:10 dilution. BSE, elk CWD (homozygous for methionine at position 132 of the prion protein [37]), and scrapie of (ARQ/ARQ genotype) were all included in the reaction at a dilution 1:10, except for BSE, which was used at a 1:5 dilution. To perform a comparison between samples before and after the amplification procedure, we took 19 µL of each reaction mixture before the serial cycles of sonication and incubation. Each cycle consisted of 20 s sonication (at an amplitude of 33, wattage range: 280–300), followed by 29 min and 40 s of incubation; we repeated this procedure 96 times (48 h).

### Evaluation of the zoonotic potential of porcine tissues using PMCA (Site 2)

Normal brain homogenates (NBH, 10% w/v), used as substrates for PMCA, were prepared from healthy, transgenic mice overexpressing human PrP with V at position 129 or human PrP with M at position 129. NBH was supplemented with 6 mM EDTA, 100 µg/mL heparin, 0.05% digitonin, and 0.01% sodium tripolyphosphate (STPP). For positive controls, 10% w/v brain homogenate from vCJD-infected or VV2 sCJD-infected patient samples were serially diluted in 129M NBH or 129V NBH, respectively, and loaded into 0.2 mL PCR tubes. NBH alone was used as negative control. For the brain and muscle porcine containing BSE, scrapie, and CWD samples, 10% w/v homogenates were added to 129V NBH. Duplicate BSE samples were added to 129M NBH. PMCA tubes containing 10 µL of sample, 90 µL of NBH substrate, and three Teflon beads were placed in a QSonica Q7000 sonicator and subjected to one round of PMCA involving cycles of incubation in a water bath at 37°C for 29 min 30 s and sonication for 30 s at ~13A for 144 cycles. Samples were subjected to 100 µg/mL PK and shaken at 650 rpm at constant 37 °C for 1 h. Digestion was stopped with 4X LDS samples buffer and boiling for 10 min. Results were visualized by SDS-PAGE and immunoblotting with the 3F4 antibody (1:10,000).

## SUPPLEMENTAL MATERIAL

Supplemental Figures mbio.01800-25-s0001.pdf Figures S1 to S3.

ASM does not own the copyrights to Supplemental Material that may be linked to, or accessed through, an article. The authors have granted ASM a non-exclusive, world-wide license to publish the Supplemental Material files. Please contact the corresponding author directly for reuse.
