# Supplementary material for: Infectious prions in brains and muscles of domestic pigs experimentally challenged with the BSE, scrapie, and CWD agents
Source: mBio. 2025 Aug 18;16(9):e01800-25. doi: 10.1128/mbio.01800-25 (PMC12421853; doi:10.1128/mbio.01800-25)
Supplement: Supplemental Figures — Figures S1 to S3. [file mbio.01800-25-s0001.pdf]

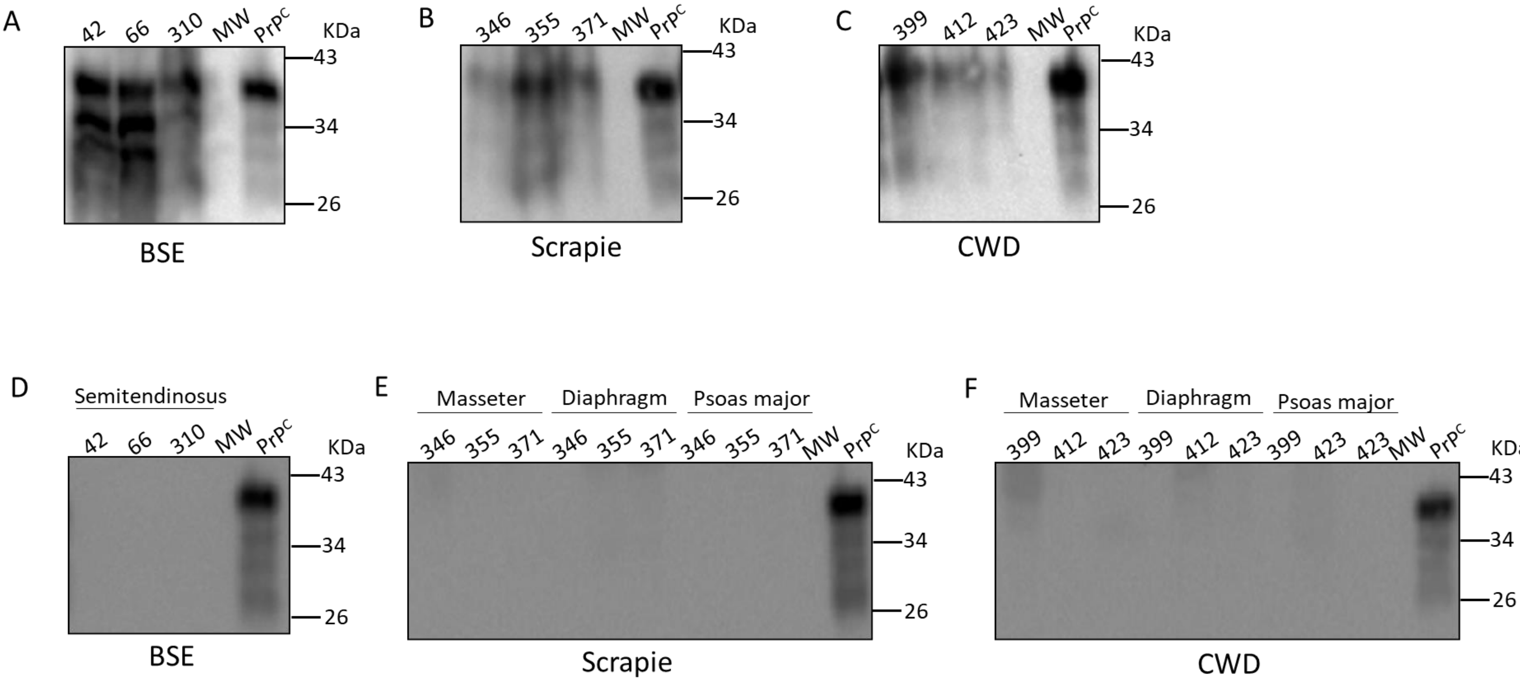

S1 Fig. Representative detection of total PrP in brain and skeletal muscle tissues of domestic pigs experimentally inoculated with BSE-, scrapie-, and CWD- prions. A) Brain homogenate (BH) from swine inoculated with C-type BSE prion (sample 42, 66, and 310). **B)** BH from pigs inoculated with US scrapie prions (sample 346, 355, and 371). **C)** BH from swine inoculated with US CWD prions (sample 399, 412, and 423). **D)** Semitendinosus muscle homogenate from domestic pigs inoculated with C-type BSE prion (sample 42, 66, and 310). **E)** Masseter, diaphragm, and psoas major muscle homogenate from pigs inoculated with US scrapie prions (sample 346, 355, and 371). **F)** Masseter, diaphragm, and psoas major muscle homogenates from pigs inoculated with US CWD prions (sample 399, 412, and 423). The total PrP was detected using the mAb 6H4 at a dilution of 1:10,000. The numbers on the top of the panels represent the sample analyzed. The numbers at the right represent molecular weight markers (in KDa). The name on the bottom of each panel depicts the prion strain used to inoculate the domestic pigs. The MW, represents the EZ-Run molecular weight ladder (Fisher Scientific, Waltham, MA, US). “PrP^C” denotes brain extracts from Tg002 mice (expressing the pig version of PrP^C) not treated with PK and used as an electrophoretic mobility control.

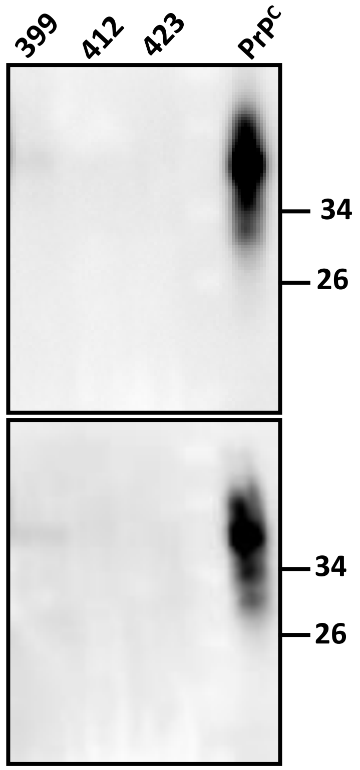

<u>S2 Fig.</u> PK-resistant PrP^{Sc} in the brain of domestic pigs experimentally challenged with CWD. Undiluted brain homogenates (10% w/v) from pigs treated with CWD prions were PK-treated and probed with the mAb 8H4 (1:10,000). All samples were tested in duplicate (upper and lower membranes). The numbers at the right represents molecular weight markers (in KDa). "PrP^C" denotes brain extracts from a Tg002 mouse (expressing the pig version of PrP^C) that was not treated with PK. This "PrP^C" sample was used as an electrophoretic mobility and antibody reactivity control.

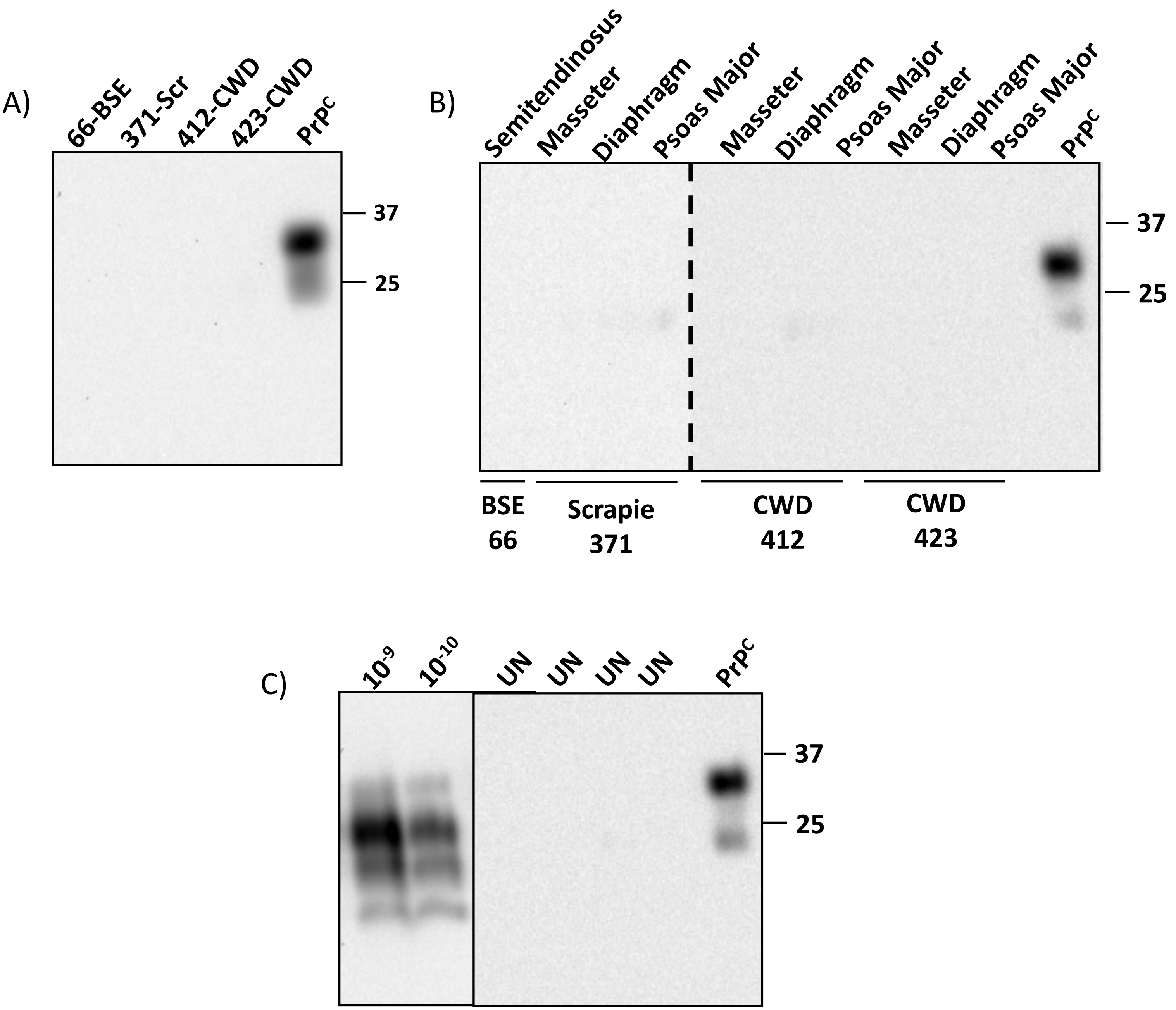

<u>S3 Fig.</u> *In vitro* misfolding of human prion protein (129V) seeded with tissues from pigs experimentally inoculated with the BSE, scrapie, and CWD agents. A) Humanized PMCA (PrP 129V) reactions seeded with brain homogenates from swine exposed to C-type BSE (ID 66), scrapie (ID 371), and CWD (IDs 412 and 423) prions. **B)** Humanized PMCA (PrP 129V) reactions seeded with skeletal muscle homogenates from swine exposed to C-type BSE (ID 66), scrapie (ID 371), and CWD (IDs 412 and 423). **C)** Technical controls consisted of seeded reactions with serial dilutions (10^{-9} and 10^{-10}) from the brain homogenate from a CJD VV2 individual. Additionally, unseeded (UN) PMCA reactions were included. PMCA products were detected using the mAb 3F4 (1:5,000). Numbers at the right of each panel represent molecular weight markers (in KDa). "PrP^C" denotes brain extracts from Tg-HuMM mice (expressing the 129M version of human PrP) not treated with PK and used as an electrophoretic mobility and antibody reactivity controls. The dashed (B) and continuous (C) lines between blots represents cropped/edited membranes.
